# Publisher Correction: Publisher correction for multiple articles

**DOI:** 10.1038/s43705-022-00206-4

**Published:** 2022-12-08

Correction to: *ISME Communications* 10.1038/s43705-021-00035-x, published online 12 July 2021;

*ISME Communications* 10.1038/s43705-021-00042-y, published online 20 August 2021;

*ISME Communications* 10.1038/s43705-021-00044-w, published online 18 October 2021;

*ISME Communications* 10.1038/s43705-021-00047-7, published online 1 September 2021;

*ISME Communications* 10.1038/s43705-021-00058-4 published online 11 October 2021

The original versions of the articles with the above DOIs were unfortunately missing the Open Access License text: This article is licensed under a Creative Commons Attribution 4.0 International License, which permits use, sharing, adaptation, distribution and reproduction in any medium or format, as long as you give appropriate credit to the original author(s) and the source, provide a link to the Creative Commons licence, and indicate if changes were made. The images or other third-party material in this article are included in the article’s Creative Commons licence, unless indicated otherwise in a credit line to the material. If material is not included in the article’s Creative Commons licence and your intended use is not permitted by statutory regulation or exceeds the permitted use, you will need to obtain permission directly from the copyright holder. To view a copy of this licence, visit http://creativecommons.org/licenses/by/4.0/. In addition, they have a copyright holdername issue as well. The affected articles are 10.1038/s43705-021-00035-x, 10.1038/s43705-021-00042-y, 10.1038/s43705-021-00044-w, 10.1038/s43705-021-00047-7, and 10.1038/s43705-021-00058-4.

We apologize for the errors. The original articles have been corrected.

